# Correction to: A standardised protocol for neuro-endoscopic lavage for post-haemorrhagic ventricular dilatation: a Delphi consensus approach

**DOI:** 10.1007/s00381-023-05866-8

**Published:** 2023-03-04

**Authors:** DOLPHIN-UK Collaborators, Kristian Aquilina, Conor Mallucci, Aswin Chari, Saniya Mediratta, Gnanamurthy Sivakumar, Greg James, Ibrahim Jalloh, John Kitchen, Matthew A. Kirkman, Patricia de Lacy, Paul Leach, Shailendra Ashok Magdum, William Dawes, William B. Lo

**Affiliations:** Department of Neurosurgery, Great Ormond Street Hospital, London, UK

**Correction to: Child's Nervous System (2022) 38:2181–2187** **https://doi.org/10.1007/s00381-022-05632-2**

In this article, authors “Saniya Mediratta” and “Aswin Chari “ should be remove in author group field but included in “DOLPHIN-UK Collaborators “ then listed at the bottom as corresponding authors.

The original article has been corrected.

