# Supplementary material for: A standardised protocol for neuro-endoscopic lavage for post-haemorrhagic ventricular dilatation: A Delphi consensus approach
Source: Childs Nerv Syst. 2022 Oct 7;38(11):2181–7. doi: 10.1007/s00381-022-05632-2 (PMC9617835; doi:10.1007/s00381-022-05632-2)
Supplement: Supplementary file 1 — Supplementary file1 (DOCX 18 KB) [file 381_2022_5632_MOESM1_ESM.docx]

*Supplementary Figure 1: Phase 1b Survey*

Achieving Consensus for Neuroendoscopic Lavage (NEL) in PHVD

*On whom should NEL be performed?*

- *All IVH*
- *Grade II+*
- *Grade III+*
- *Grade IV only*

*When should NEL be performed?*

- *Soon after diagnosis of IVH*
- *On development of PHVD: Before VI crosses 97^th^ centile*
- *On development of PHVD: When VI crosses 87^th^ centile*
- *On development of PHVD: When VI crosses 97^th^ centile + 4mm*
- *Other*

*Should NEL be delayed until the infant is stable from a systematic perspective (ventilation, inotropes, NEC, sepsis, etc)*

- *Yes*
- *No*

*What endoscope can be used?*

- *Rigid*
- *Flexible*
- *Either*

*Which ventricle should be entered?*

- *Ventricle with higher clot burden*
- *Ventricle with lower clot burden*
- *Always the right ventricle*
- *Always the left ventricle*
- *Does not matter*

*Where should the ventricle be cannulated?*

- *Frontal*
- *Parietal*
- *Other*

*How much wash should be used during lavage?*

- *1 litre*
- *2 litres*
- *Does not matter*
- *Other*

*What wash should be used for lavage?*

- *Artificial CSF*
- *Ringer’s lactate*
- *Plasmalyte*
- *Does not matter*
- *Other*

*What should be the endpoint of the lavage?*

- *When a certain volume of wash has been used*
- *When a certain amount of time has elapsed*
- *When the effluent fluid is clear*
- *When all of the clots have gone*
- *Other*

*What other steps would you include as part of NEL? (Select all that apply)*

- *Dislodge clots*
- *Septostomy*
- *Third ventriculostomy*
- *Choroid plexus coagulation*
- *Other*

*What should be performed at the end of NEL*

- *Ventricular access device*
- *Ventriculosubgaleal shunt*
- *Either temporising device*
- *No temporising device*

*When should prophylactic antibiotics be given? (Select all that apply)*

- *At induction*
- *At the end of the procedure – intrathecally*
- *Post-operatively for 24-48 hours*

*Any other comments about procedure standardisation?*

*Supplementary Figure 2: Consensus Participants*

| Participant Name | Role | Institution |
| --- | --- | --- |
| Conor Mallucci | Consultant Paediatric Neurosurgeon | Alder Hey Children’s Hospital, Liverpool |
| Gnanamurthy Sivakumar | Consultant Paediatric Neurosurgeon | Leeds Children’s Hospital, Leeds |
| Greg James | Consultant Paediatric Neurosurgeon | Great Ormond Street Hospital, London |
| Ibrahim Jalloh | Consultant Paediatric Neurosurgeon | Addenbrooke’s Hospital, Cambridge |
| John Kitchen | Consultant Paediatric Neurosurgeon | Royal Manchester Children’s Hospital, Manchester |
| Kristian Aquilina | Consultant Paediatric Neurosurgeon | Great Ormond Street Hospital, London |
| Matthew Kirkman | Consultant Paediatric Neurosurgeon | Queen’s Medical Centre, Nottingham |
| Patricia de Lacy | Consultant Paediatric Neurosurgeon | Sheffield Children’s Hospital, Sheffield |
| Paul Leach | Consultant Paediatric Neurosurgeon | University Hospital of Wales, Cardiff |
| Shailendra Ashok Magdum | Consultant Paediatric Neurosurgeon | John Radcliffe Hospital, Oxford |
| William Dawes | Consultant Paediatric Neurosurgeon | Alder Hey Children’s Hospital, Liverpool |
| William Lo | Consultant Paediatric Neurosurgeon | Birmingham Children’s Hospital, Birmingham |
